# The crosstalk of HDAC3, microRNA-18a and ADRB3 in the progression of heart failure

**DOI:** 10.1186/s13578-020-00523-y

**Published:** 2021-02-06

**Authors:** Jingtao Na, Haifeng Jin, Xin Wang, Kan Huang, Shuang Sun, Qiang Li, Wenting Zhang

**Affiliations:** 1 Department of Cardiology, The Third Affiliated Hospital of Qiqihar Medical University, No. 27, Taishun Street, Tiefeng District, Qiqihar, 161099 Heilongjiang Province P.R. China; 2 Department of Anatomy, Qiqihar Medical University, Qiqihar, 161006 P.R. China; 3 Department of Clinical Pharmacy, The Third Affiliated Hospital of Qiqihar Medical University, Qiqihar, 161099 P.R. China

**Keywords:** Heart failure, Fibrosis, Hypertrophy, microRNA-18a, HDAC3, ADRB3

## Abstract

**Background:**

Heart failure (HF) is a clinical syndrome characterized by left ventricular dysfunction or elevated intracardiac pressures. Research supports that microRNAs (miRs) participate in HF by regulating  targeted genes. Hence, the current study set out to study the role of HDAC3-medaited miR-18a in HF by targeting ADRB3.

**Methods:**

Firstly, HF mouse models were established by ligation of the left coronary artery at the lower edge of the left atrial appendage, and HF cell models were generated in the cardiomyocytes, followed by ectopic expression and silencing experiments. Numerous parameters including left ventricular posterior wall dimension (LVPWD), interventricular septal dimension (IVSD), left ventricular end diastolic diameter (LVEDD), left ventricular end systolic diameter (LVESD), left ventricular ejection fraction (LVEF), left ventricular fractional shortening (LVFS), left ventricular systolic pressure (LVSP), left ventricular end diastolic pressure (LEVDP), heart rate (HR), left ventricular pressure rise rate (+ dp/dt) and left ventricular pressure drop rate (-dp/dt) were measured in the mice. In addition, apoptosis in the mice was detected by means of TUNEL staining, while RT-qPCR and Western blot analysis were performed to detect miR-18a, HDAC3, ADRB3, cMyb, MMP-9, Collagen 1 and TGF-β1 expression patterns. Dual luciferase reporter assay validated the targeting relationship between ADRB3 and miR-18a. Cardiomyocyte apoptosis was determined by means of flow cytometry.

**Results:**

HDAC3 and ADRB3 were up-regulated and miR-18a was down-regulated in HF mice and cardiomyocytes. In addition, HDAC3 could reduce the miR-18a expression, and ADRB3 was negatively-targeted by miR-18a. After down-regulation of HDAC3 or ADRB3 or over-expression of miR-18a, IVSD, LVEDD, LVESD and LEVDP were found to be decreased but LVPWD, LVEF, LVFS, LVSP, + dp/dt, and −dp/dt were all increased in the HF mice, whereas fibrosis, hypertrophy and apoptosis of HF cardiomyocytes were declined.

**Conclusion:**

Collectively, our findings indicate that HDAC3 silencing confers protection against HF by inhibiting miR-18a-targeted ADRB3.

## Background

Heart failure (HF) is a universal health epidemic that affects over 26 million people globally, the incidence of which is estimated to increase with ageing populations [1]. The hallmark characteristics of HF are abnormal left ventricular and/or elevated intracardiac pressures [2]. Pathologically, HF is classified as a condition where the heart is unable to provide adequate oxygen supply to support the basic metabolism of cardiac tissues [3]. Patients with chronic HF experience deteriorating health status in spite of receiving conventional therapy, which elicits that the clinical drug trials and preserved ejection fraction in patients with HF have been futile in improving mortality [4, 5]. Fortunately, the hard-done work of our peers has elicited an association between aberrant expression of microRNAs (miRs) with HF and cardiac re-modelling [6].

Accumulating studies have identified the critical importance of miRs in various processes such as the initiation, progression and perpetuation of HF. Moreover, miRs were recently highlighted to hold great potential as viable biomarkers for the diagnosis and prognosis of HF [7]. More importantly, the declining expression of various circulating miRs have been closely related with worsening acuity of HF [8]. One such miR, namely miR-18a, has been closely-associated with HF by numerous authors. For instance, lower levels of miR-18a-5p were associated with recurrent rehospitalizations due to atherosclerotic and cardiovascular disease in HF patients [9]. In addition, miR-18a-5p was negatively-associated with biomarkers imperative to worsening outcomes in HF patients [10]. Furthermore, an existing study identified that histone deacetylase 3 (HDAC3) can inhibit the expression of miR-18a [11]. HDACs are a type of principal epigenetic regulatory enzymes, which possess the ability to alleviate cardiac hypertrophy and cardiac fibrosis [12]. Reports have also documented the vital functionality of HDACs in the progression of HF. For example, decreased expression of HDAC3 was associated with improved cardiac function of mice with left ventricular HF [13]. Additionally, the loss of the epigenomic modifier HDAC3 can also precipitate dietary death by attenuating the metabolism of the cardiac mitochondria responding to homeostatic changes of the nutritional environment [14]. Initial bio-prediction findings in our study indicated the adrenergic-receptor β3 (ADRB3) gene as a target of miR-18a. As a member of the obesity genes, ADRB3 mediates the energy balance by stimulating lipolysis and thermogenesis [15]. Also, ADRB3 has been identified to be closely associated with cardiac diseases like HF. For example, ADRB3 could facilitate the development of left ventricular diastolic dysfunction, which is a critical cause of HF [16].

On the basis of the literature, we hypothesized that HDAC3, miR-18a and ADRB3 were potential participants in the progression of HF. Therefore, the current study was performed to explore the detailed correlation between miR-18a, HDAC3 and ADRB3 and their underlying mechanism in the progression of HF.

## Results

### miR-18a was under-expressed in HF mice and miR-18a overexpression could improve HF in mice

**Fig. 1:**
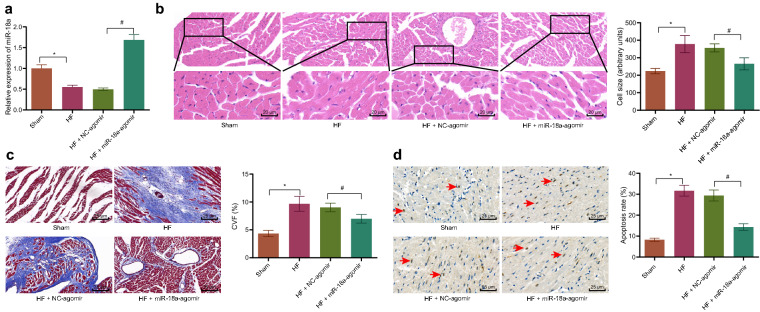
miR-18a is poorly expressed in HF and miR-18a overexpression alleviates HF in mice. Sham-operated mice were used as controls, and HF mice were treated or not treated with NC-agomir or miR-18a-agomir. **a** RT-qPCR detection of the miR-18a expression pattern in the mouse heart tissue normalized to U6. **b** Cardiomyocyte size in the myocardial tissues observed under HE staining (400×). **c** The degree of CVF in the mouse heart tissue detected by Masson staining (200×). **d** The apoptosis of mouse heart cells determined by TUNEL staining (200×). * *p* < 0.05 *vs.* sham-operated mice; # *p* < 0.05 *vs.* the HF mice injected with NC-agomir. n = 10. The measurement data (mean ± standard deviation) were compared by the *t* test. One-way ANOVA was utilized for comparisons among multiple groups, and the Tukey’s post-hoc test was employed for intra-group pairwise comparison

Firstly, mouse models of HF were established in order to investigate whether miR-18a was involved in alleviation of HF. The results of echocardiography demonstrated that LVP, LVEF and LVFS were decreased in HF mice, while IVSD, LVEDD and LVESD were increased. Meanwhile, treatment with miR-18a-agomir brought about contradictory findings (Table 1). In addition, hemodynamics results illustrated that LVSP, dp/dt and -dp/dt were reduced, and LEVDP was elevated in HF mice, while these findings could be countered by treatment with miR-18a-agomir (Table 2). RT-qPCR results manifested that miR-18a expression was reduced in the heart tissues of HF mice, while being elevated in the heart tissues of HF mice injected with miR-18a-agomir (Fig. 1a). Additionally, HE staining demonstrated the presence of enlarged cardiomyocytes in HF mice, which could be annulled following treatment with miR-18a-agomir (Fig. 1b). Also, Masson staining demonstrated that CVF was increased in mice after HF model establishment, while the opposite findings were noted in the heart tissues of HF mice treated with miR-18a-agomir (Fig. 1c). TUNEL staining further revealed that apoptosis was elevated in the heart tissues of HF mice, while being reduced following treatment with miR-18a-agomir (Fig. 1d). Together, the aforementioned results indicated that miR-18a was poorly-expressed in HF mouse models and miR-18a over-expression could improve HF in mice.

**Table 1:** Echocardiographic results of mice after miR-18a inhibition

Group\parameter	LVPWD (mm)	IVSD (mm)	LVEDD (mm)	LVESD (mm)	LVEF (%)	LVFS (%)
Sham	1.48 ± 0.18	0.51 ± 0.05	3.74 ± 0.49	1.77 ± 0.19	51.87 ± 8.98	52.67 ± 6.23
HF	1.11 ± 0.13*	0.69 ± 0.07*	4.71 ± 0.61*	3.73 ± 0.29*	17.55 ± 1.85*	11.82 ± 1.54*
HF + NC-agomir	1.02 ± 0.09	0.72 ± 0.11	4.52 ± 0.45	3.91 ± 0.41	16.22 ± 1.66	10.32 ± 1.28
HF + miR-18a-agomir	1.54 ± 0.08#	0.59 ± 0.07#	3.89 ± 0.16#	1.91 ± 0.28#	50.79 ± 7.53#	49.47 ± 4.94#

^*^ *p* < 0.05 *vs.* sham-operated mice; # *p* < 0.05 *vs.* the HF mice injected with NC-agomir. One-way ANOVA was utilized for comparisons among multiple groups, and Tukey’s post-hoc test was employed for intra-group pairwise comparison

**Table 2:** Hemodynamic indicators of mice after miR-18a inhibition

Group\parameter	LVSP(mm Hg)	LEVDP(mm Hg)	dp/dt(mm Hg/s)	-dp/dt(mm Hg/s)
Sham	112.27 ± 8.13	7.62 ± 0.38	8257.61 ± 976.83	9848.71 ± 856.87
HF	86.31 ± 5.61*	25.91 ± 1.91*	6336.27 ± 550.42*	5563.47 ± 623.61*
HF + NC-agomir	79.28 ± 6.34	24.66 ± 1.03	6762.82 ± 535.68	5442.23 ± 403.98
HF + miR-18a-agomir	97.86 ± 12.78#	8.46 ± 0.78#	8019.39 ± 814.23#	9659.33 ± 883.61#

^*^ *p* < 0.05 *vs.* sham-operated mice; # *p* < 0.05 *vs.* the HF mice injected with NC-agomir. One-way ANOVA was utilized for comparisons among multiple groups, and Tukey’s post-hoc test was employed for intra-group pairwise comparison

### miR-18a could inhibit fibrosis, cardiac hypertrophy, and apoptosis in HF cardiomyocytes

**Fig. 2:**
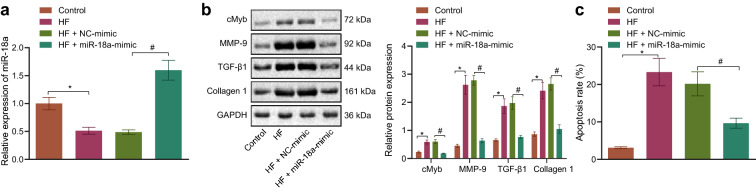
miR-18a suppresses fibrosis, cardiac hypertrophy and apoptosis in HF cardiomyocytes. Normal cardiomyocytes were used as controls, and HF cardiomyocytes were transfected or not transfected with NC-mimic or miR-18a mimic. **a** miR-18a expression pattern in the mouse cardiomyocytes detected by RT-qPCR normalized to U6. **b** Western blot analysis of the expression patterns of cMyb, MMP-9, Collagen 1 and TGF-β1 protein in mouse cardiomyocytes normalized to GAPDH. **c** Apoptosis of mouse cardiomyocytes determined by flow cytometry. * *p* < 0.05 *vs.* control cardiomyocytes. # *p* < 0.05*vs.* HF cardiomyocytes transfected with NC-mimic. n = 10. The measurement data (mean ± standard deviation) were compared by the *t* test. The experiment was conducted 3 times independently. One-way ANOVA was utilized for comparisons among multiple groups, and the Tukey’s post-hoc test was employed for intra-group pairwise comparison

To further elucidate the role of miR-18a in cardiomyocytes, mouse HF cell models were established with the help of mouse cardiomyocytes. Subsequent RT-qPCR results demonstrated that miR-18a expression was diminished in HF cell models, while treatment with miR-18a mimic inverted this finding (Fig. 2a). Cardiomyocyte imaging results further illustrated that cardiomyocytes had increased in size following HF cell model construction, while the opposite was true for miR-18a mimic treatment (Additional file 1: Fig. S1A). Western blot analysis displayed cMyb, MMP-9, Collagen 1 and TGF-β1 expression was all significantly elevated in HF cell models, while these trends were reversed by miR-18a mimic treatment (Fig. 2b). These findings suggested that myocardial fibrosis was exacerbated in HF cell models, and could be inhibited after over-expression of miR-18a. Meanwhile, the results of flow cytometry illustrated that apoptosis was also elevated in HF cell models, while being inhibited after miR-18a mimic treatment (Fig. 2c). Collectively, these findings indicated that cardiomyocyte fibrosis, cardiac hypertrophy and apoptosis in HF cardiomyocytes could be repressed by miR-18a over-expression.

### miR-18a negatively-targeted ADRB3 in mouse cardiomyocytes

**Fig. 3:**
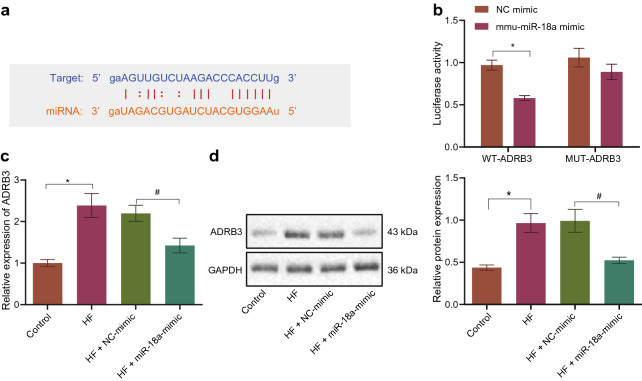
ADRB3 is negatively targeted by miR-18a. **a** Binding sites between miR-18a and ADRB3-3′UTR. **b** Targeting relationship between miR-18a and ADRB3 evaluated by dual luciferase reporter assay (mouse), * *p* < 0.05 vs. the cells transfected with NC-mimic. **c** RT-qPCR detection of the mRNA expression pattern of ADRB3 in HF cardiomyocytes after miR-181a overexpression normalized to GAPDH. **d** Western blot analysis of detected the protein expression pattern of ADRB3in HF cardiomyocytes after miR-181a overexpression normalized to GAPDH. * *p* < 0.05 vs. control cardiomyocytes. # *p* < 0.05 vs. HF cardiomyocytes transfected with NC-mimic. The data results were measurement data and expressed as mean ± standard deviation. n = 10. Independent sample *t*-test was adopted to compare the data between two groups. One-way ANOVA was utilized for comparisons among multiple groups, and Tukey’s post-hoc test was employed for intra-group pairwise comparison. The experiment was conducted 3 times independently

To further investigate the downstream mechanisms regulated by miR-18a, starBase was adopted to predict the downstream target genes of miR-18a, and the findings indicated the presence of a binding site between miR-18a and ADRB3 (Fig. 3a). Subsequent dual luciferase reporter assay results demonstrated a decrease in the luciferase activity of WT-ADRB3 after transfection with miR-18a mimic, while no significant difference was observed in the luciferase activity of the mutant ADRB3 3′UTR (Fig. 3b). Also, the mRNA and protein expression patterns of ADRB3 were found to be elevated in the mouse cardiomyocytes of HF models, while the opposite was true following miR-18a mimic treatment (Fig. 3c, d). Altogether, these findings indicated that ADRB3 was a direct target gene of miR-18a, and miR-18a inhibited the expression of ADRB3 in mouse cardiomyocytes.

### miR-18a could inhibit fibrosis, cardiac hypertrophy and apoptosis by targeting ADRB3 in HF cardiomyocytes

**Fig. 4:**
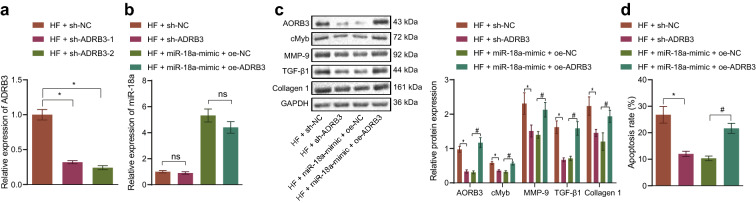
miR-18a targets ADRB3 to repress cardiomyocyte fibrosis, cardiac hypertrophy and apoptosis of HF cardiomyocyte. **a** RT-qPCR detecting ADRB3 mRNA level in mouse cardiomyocytes transfected with sh-ADRB3-1 or sh-ADRB3-2 normalized to GAPDH. HF cardiomyocytes were transfected with sh-NC, sh-ADRB, miR-18a-mimic + oe-NC or miR-18a-mimic + oe-ADRB3. **b** miR-18a expression pattern in mouse cardiomyocytes detected by RT-qPCR normalized to U6. **c** Western blot analysis of the expression pattern of ADRB3, cMyb, MMP-9, Collagen 1 and TGF-β1 protein in mouse cardiomyocytes normalized to GAPDH. **d** Flow cytometry detection of the apoptosis of mouse cardiomyocytes. * *p* < 0.05 *vs.* HF cells transfected with sh-NC, # *p* < 0.05 *vs.* HF cells transfected with miR-18a-mimic + oe-NC. The data were measurement data and expressed as mean ± standard deviation. One-way ANOVA was utilized for comparisons among multiple groups, and Tukey’s post-hoc test was employed for intra-group pairwise comparison. The experiment was conducted 3 times independently

To further investigate the involvement of miR-18a in the development of HF via ADRB3 inhibition, two silencing sequences of ADRB3 were prepared in this study. RT-qPCR results documented that after transfection with sh-ADRB3-1 or sh-ADRB3-2, ADRB3 expression was lowered in the cardiomyocytes, and the cells transfected with sh-ADRB3-1 showing the better silencing efficiency were selected for subsequent experimentation (Fig. 4a). In addition, RT-qPCR results further demonstrated that miR-18a expression was increased in HF cells transfected with miR-18a-mimic + oe-NC or miR-18a-mimic + oe-ADRB3 (Fig. 4b). Meanwhile, Western blot analysis illustrated that ADRB3, cMyb, MMP-9, Collagen 1 and TGF-β1 expression patterns were all decreased following transfection with sh-ADRB3 or miR-18a mimic, while over-expressing miR-18a and ADRB3 negated the aforementioned effects (Fig. 4c). Also, the results of cardiomyocyte imaging exhibited an evident decrease in cardiomyocyte size in the presence of sh-ADRB3 or miR-18a mimic, while the corresponding effects of miR-18a mimic on cardiomyocyte size could be normalized by over-expression of miR-18a and ADRB3 (Additional file 1: Fig. S1B). Flow cytometry results suggested that inhibition of ADRB3 or over-expression of miR-18a brought about a decline in cardiomyocyte apoptosis, while these effects could be reversed by up-regulating miR-18a and ADRB3 (Fig. 4d). Overall, these findings suggested that miR-18a targeting ADRB3 inhibited cardiomyocyte fibrosis, cardiac hypertrophy and apoptosis.

### miR-18a could alleviate HF by inhibiting ADRB3 in mice

**Fig. 5:**
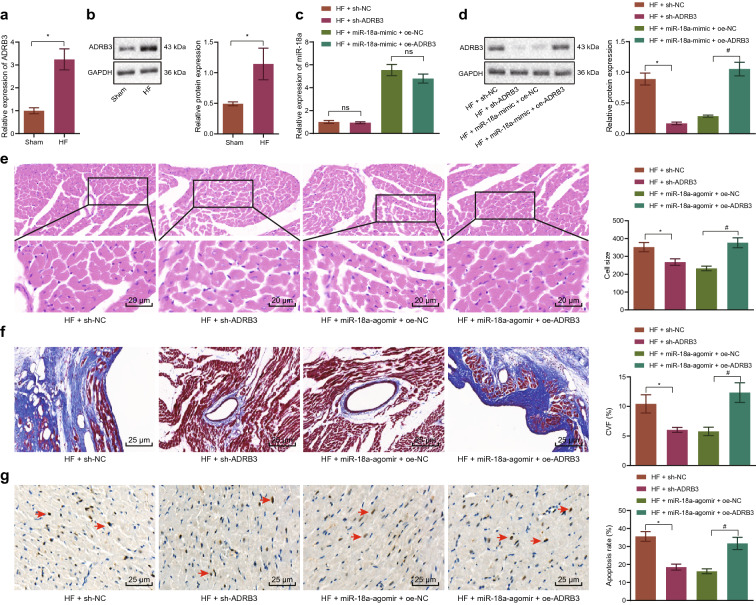
miR-18a alleviates HF by inhibiting ADRB3 in mice. **a** RT-qPCR detection of the mRNA expression of ADRB3 in mouse heart tissue after HF mice model establishment normalized to GAPDH. **b** Western blot analysis of the protein expression pattern of ADRB3 in mouse heart tissue after HF mice model establishment normalized to GAPDH. * *p* < 0.05 *vs.* sham-operated mice. HF mice were treated with sh-NC, sh-ADRB, miR-18a-agomir + oe-NC or miR-18a-agomir + oe-ADRB3. **c** RT-qPCR detection of the expression pattern of miR-18a in mouse heart tissue normalized to U6. **d** Western blot analysis of the expression pattern of ADRB3 in mouse heart tissue normalized to GAPDH. **e** Cardiomyocyte size in the myocardial tissues observed by HE staining (400×). **f** The degree of CVF (200×) in mouse heart tissues detected by Masson staining. **g** The apoptosis of mouse heart cells determined by TUNEL staining (200×). * *p* < 0.05 *vs.* HF mice treated with sh-NC. # *p* < 0.05 vs. HF mice treated with miR-18a-agomir + oe-NC. The data were measurement data and expressed as mean ± standard deviation. n = 10. One-way ANOVA was utilized for comparisons among multiple groups, and Tukey’s post-hoc test was employed for intra-group pairwise comparison

To further explore the role of ADRB3 in HF mice and whether miR-18a improved HF by inhibiting ADRB3 expression in mice, miR-18a was over-expressed and ADRB3 was altered in HF mice. Subsequently, ADRB3 expression was found to be increased in HF mice (Fig. 5a, b). In addition, miR-18a expression was elevated in HF mice in response to miR-18a-agomir + oe-NC or miR-18a-agomir + oe-ADRB3 (Fig. 5c). Meanwhile, HF mice treated with miR-18a-agomir exhibited a decline in ADRB3 expression in mouse myocardium, which could be annulled by over-expressing ADRB3 (Fig. 5d). Also, increased LVPWD, LVEF and LVFS and decreased IVSD, LVEDD and LVESD were noted in HF mice treated with miR-18a-agomir or sh-ADRB3, which could be countered by combination treatment with miR-18a-agomir and oe-ADRB3 (Table 3). On the other hand, LVSP, dp/dt and -dp/dt were increased, while LEVDP was decreased in HF mice in the presence of miR-18a-agomir or sh-ADRB3, whereas the opposite findings were noted after simultaneous treatment with miR-18a-agomir and oe-ADRB3 (Table 4). HE staining illustrated a reduction in cardiomyocyte size (Fig. 5e), CVF (Fig. 5f) and apoptosis (Fig. 5g) in HF mice treated with miR-18a-agomir or sh-ADRB3, which could be obliterated by concurrent treatment with miR-18a-agomir and oe-ADRB3. To sum up, these findings indicated that miR-18a contributed to the improvement of HF through inhibition of ADRB3 in mice.

**Table 3:** Echocardiographic results of mice after alteration of ADRB3 and overexpression of miR-18a

Group\parameter	LVPWD (mm)	IVSD (mm)	LVEDD (mm)	LVESD (mm)	LVEF (%)	LVFS (%)
HF + sh-NC	1.26 ± 0.14	0.74 ± 0.08	4.57 ± 0.58	3.87 ± 0.49	15.33 ± 1.01	15.13 ± 1.23
HF + sh-ADRB3	1.75 ± 0.19*	0.51 ± 0.07*	3.70 ± 0.31*	2.83 ± 0.21 *	23.48 ± 2.01*	24.13 ± 2.54*
HF + miR-18a-agomir + oe-NC	1.84 ± 0.17	0.57 ± 0.06	3.54 ± 0.31	2.66 ± 0.24	24.83 ± 1.59	26.32 ± 2.28
HF + miR-18a-agomir + oe-ADRB3	1.31 ± 0.08#	0.82 ± 0.09 #	4.37 ± 0.50 #	3.67 ± 0.42 #	15.99 ± 2.34 #	16.21 ± 1.14 #

^*^ *p* < 0.05 *vs.* HF mice treated with sh-NC. # *p* < 0.05 *vs.* HF mice treated with miR-18a-agomir + oe-NC. One-way ANOVA was utilized for comparisons among multiple groups, and Tukey’s post-hoc test was employed for intra-group pairwise comparison

**Table 4:** Hemodynamic indicators in mice after alteration of ADRB3 and overexpression of miR-18a

Group\parameter	LVSP (mm Hg)	LEVDP (mm Hg)	dp/dt (mm Hg/s)	dp/dt (mm Hg/s)
HF + sh-NC	81.56 ± 8.94	19.02 ± 2.14	6587.94 ± 545.34	5253.77 ± 667.49
HF + sh-ADRB3	118.69 ± 10.78 *	11.84 ± 1.07*	9453.73 ± 787.51 *	9982.83 ± 940.21 *
HF + miR-18a-agomir + oe-NC	113.78 ± 18.06	12.61 ± 1.44	9632.01 ± 810.12	9791.16 ± 917.24
HF + miR-18a-agomir + oe-ADRB3	95.61 ± 8.08 #	18.37 ± 2.09 #	6964.48 ± 545.33 #	5723.26 ± 346.42 #

^*^ *p* < 0.05 *vs.* HF mice treated with sh-NC. # *p* < 0.05 *vs.* HF mice treated with miR-18a-agomir + oe-NC. One-way ANOVA was utilized for comparisons among multiple groups, and Tukey’s post-hoc test was employed for intra-group pairwise comparison

### HDAC3 silencing could up-regulate miR-18a to reduce ADRB3 expression, consequently suppressing fibrosis, hypertrophy and apoptosis of HF cardiomyocytes

**Fig. 6:**
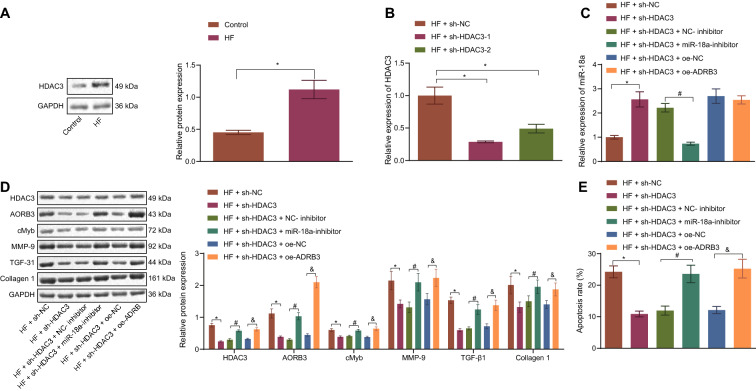
HDAC3 silencing activates miR-18a to decrease the ADRB3 expression pattern, thus inhibiting cardiomyocyte fibrosis, hypertrophy and apoptosis. **a** Western blot analysis of HDAC3 expression pattern in HF cardiomyocytes normalized to GAPDH. **b** RT-qPCR detection of HDAC3 expression pattern in HF cardiomyocytes transfected with sh-HDAC3-1 or sh-HDAC3-2normalized to GAPDH. HF cardiomyocytes were transfected with sh-NC, sh-HDAC3, NC-inhibitor + sh-HDAC3, miR-18a + sh-HDAC3, sh-HDAC3 + oe-NC or sh-HDAC3 + oe-ADRB3. **c** RT-qPCR detection of miR-18a expression pattern in mouse cardiomyocytes normalized to U6. **d** Western blot analysis of the expression patterns of ADRB3, cMyb, MMP-9, Collagen 1 and TGF-β1 protein in mouse cardiomyocytes normalized to GAPDH. **e** Flow cytometry detection of the apoptosis of mouse cardiomyocytes. * *p* < 0.05 *vs.* HF cardiomyocytes transfected with sh-NC. # *p* < 0.05 *vs.* HF cardiomyocytes transfected with NC-inhibitor + sh-HDAC3. & *p* < 0.05 *vs.* HF cardiomyocytes transfected with sh-HDAC3 + oe-NC. n = 10. The data were measurement data and expressed as mean ± standard deviation. The *t* test was adopted for comparison between the two groups and one-way ANOVA was used for comparison among multiple groups, and the Tukey’s post-hoc test was employed for intra-group pairwise comparison. The experiment was conducted 3 times independently

Findings of Western blot analysis demonstrated that HDAC3 levels were elevated in cardiomyocytes after HF model establishment (Fig. 6a). RT-qPCR further revealed that transfection with sh-HDAC3-1 or sh-HDAC3-2 brought about a reduction in HDAC3 expression in cardiomyocytes (Fig. 6b). Moreover, sh-HDAC3 was found to trigger miR-18a upregulation in HF cardiomyocytes, which was inverted following combination treatment with miR-18a inhibitor and sh-HDAC3 (Fig. 6c). As illustrated by the cardiomyocyte imaging results, silencing HDAC3 precipitated a decrease in cardiomyocyte size, while the opposite was true for silencing miR-18a and HDAC3 or silencing HDAC3 and over-expressing ADRB3 (Additional file 1: Fig. S1C). Furthermore, HDAC3, ADRB3, cMyb, MMP-9, Collagen 1 and TGF-β1 expression levels were found to be lowered following HDAC3 silencing, and could be neutralized after treatment with miR-18a inhibitor and sh-HDAC3 or sh-HDAC3 and oe-ADRB3 (Fig. 6d). Flow cytometry results demonstrated that silencing of HDAC3 diminished cardiomyocytes apoptosis, which was normalized by miR-18a inhibitor and sh-HDAC3 or sh-HDAC3 and oe-ADRB3 (Fig. 6e). Together, the aforementioned results demonstrated that HDAC3 silencing down-regulated ADRB3 to repress the fibrosis, hypertrophy and apoptosis by activating miR-18a in HF cardiomyocytes.

### HDAC3 silencing activated miR-18a to suppress ADRB3, thereby attenuating HF in mice

**Fig. 7:**
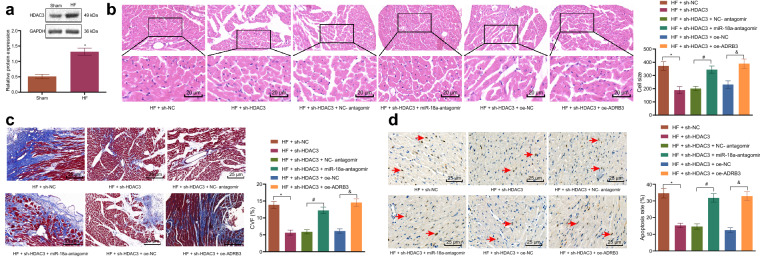
HDAC3 downregulation attenuates HF by suppressing miR-18a-targeted ADRB3 in mice. **a** Western blot analysis of the expression pattern of HDAC3 in myocardial tissues of HF mice normalized to GAPDH. HF mice were treated with sh-NC, sh-HDAC3, sh-HDAC3 + NC-antagomir, sh-HDAC3 + miR-18a-antagomir, sh-HDAC3 + oe-NC orsh-HDAC3 + oe-ADRB3. **b** Cardiomyocyte size in myocardial tissues observed by HE staining (400×). **c** The degree of CVF (200×) in mouse heart tissue detected by Masson staining. **d** The apoptosis of mouse heart cells determined by TUNEL staining (200×). * *p* < 0.05 vs. HF mice treated with sh-NC. # *p* < 0.05 vs. HF mice treated with sh-HDAC3 + NC-antagomir. &* p* < 0.05 vs. HF mice treated with sh-HDAC3 + oe-NC. The data results were measurement data and expressed as mean ± standard deviation. n = 10. Independent sample *t*-test was adopted to compare the data between two groups. One-way ANOVA was utilized for comparisons among multiple groups, and Tukey’s post-hoc test was employed for intra-group pairwise comparison

Lastly, in order to explore whether HDAC3 promoted the ADRB3 expression and aggravated HF by inhibiting miR-18a in vivo, HDAC3, miR-18a and ADRB3 expression was altered in HF mice. Echocardiographic results demonstrated that LVPWD, LVEF and LVFS were increased, and IVSD, LVEDD and LVESD were decreased in HF mice after HDAC3 inhibition, while these effects could be reversed by subsequent treatment with miR-18a-antagomir or oe-ADRB3 (Table 5). Moreover, HDAC3 silencing was found to elevate LVSP, dp/dt and -dp/dt, but reduced LEVDP in HF mice, which was negated by additional treatment with miR-18a-antagomir or oe-ADRB3 (Table 6). Meanwhile, Western blot analysis displayed that HDAC3 levels were up-regulated in the heart tissues of HF mice (Fig. 7a). Also, an increase in miR-18a expression (Additional file 2: Fig. S2A) and a reduction in ADRB3 expression (Additional file 2: Fig. S2B) were observed in HF mice treated with sh-HDAC3, which could be abrogated following combination treatment with miR-18a-antagomir or oe-ADRB3. Furthermore, the results of immunohistochemistry illustrated that the expression levels of HDAC3 and ADRB3 were significantly decreased after HDAC3 silencing, which was restored by additional treatment with miR-18a-antagomir or oe-ADRB3. In situ hybridization showed that the expression levels of miR-18a were significantly increased in mouse cardiomyocytes after HDAC3 was inhibited, and additional treatment with the miR-18a-antagomir or oe-ADRB3 annulled these findings (Additional file 2: Fig. S2C).

**Table 5:** Echocardiographic results of mice after inhibition of HDAC3 and miR-18a and overexpression of ADRB3

Group\parameter	LVPWD (mm)	IVSD (mm)	LVEDD (mm)	LVESD (mm)	LVEF (%)	LVFS (%)
HF + sh-NC	1.31 ± 0.16	0.81 ± 0.09	4.42 ± 0.54	3.91 ± 0.45	15.47 ± 1.78	11.31 ± 1.33
HF + sh-HDAC3	1.88 ± 0.19 *	0.54 ± 0.07 *	3.79 ± 0.41 *	2.57 ± 0.23 *	31.97 ± 4.49 *	32.13 ± 1.84 *
HF + sh-HDAC3 + NC-antagomir	1.96 ± 0.22	0.63 ± 0.07	4.01 ± 0.32	2.74 ± 0.20	31.44 ± 3.83	30.68 ± 2.68
HF + sh-HDAC3 + miR-18a-antagomir	1.63 ± 0.14 #	0.96 ± 0.11 #	4.88 ± 0.43 #	3.68 ± 0.34 #	24.68 ± 2.43 #	24.39 ± 2.17 #
HF + sh-HDAC3 + oe-NC	1.82 ± 0.21	0.61 ± 0.07	3.87 ± 0.32	2.60 ± 0.21	32.80 ± 3.42	29.97 ± 2.19
HF + sh-HDAC3 + oe-ADRB3	1.56 ± 0.17 &	0.92 ± 0.09 &	4.53 ± 0.43 &	3.90 ± 0.34 &	13.96 ± 1.26 &	14.57 ± 1.14 &

^*^ *p* < 0.05 *vs.* HF mice treated with sh-NC. # *p* < 0.05 *vs.* HF mice treated with sh-HDAC3 + NC-antagomir. & *p* < 0.05 *vs.* HF mice treated with sh-HDAC3 + oe-NC. One-way ANOVA was utilized for comparisons among multiple groups, and Tukey’s post-hoc test was employed for intra-group pairwise comparison

**Table 6:** Hemodynamic indicators of mice after inhibition of HDAC3 and miR-18a and overexpression of ADRB3

Group\parameter	LVSP (mm Hg)	LEVDP (mm Hg)	dp/dt (mm Hg/s)	-dp/dt(mm Hg/s)
HF + sh-NC	78.87 ± 8.94	19.97 ± 2.14	6815.39 ± 545.34	5739.87 ± 877.31
HF + sh-HDAC3	98.69 ± 13.58 *	11.83 ± 1.57 *	9223.98 ± 987.21 *	9622.24 ± 696.58 *
HF + sh-HDAC3 + NC-antagomir	106.78 ± 18.56	12.56 ± 1.03	9552.43 ± 790.42	9301.86 ± 917.94
HF + sh-HDAC3 + miR-18a-antagomir	85.61 ± 7.07 #	18.37 ± 2.54 #	6998.23 ± 755.53 #	5696.38 ± 386.52 #
HF + sh-HDAC3 + oe-NC	99.78 ± 14.06	10.84 ± 1.28	9467.76 ± 876.82	9805.54 ± 817.53
HF + sh-HDAC3 + oe-ADRB3	81.42 ± 7.68 &	21.62 ± 2.79 &	6724.83 ± 545.39&	5443.52 ± 786.02 &

^*^ *p* < 0.05 *vs.* HF mice treated with sh-NC. # *p* < 0.05 *vs.* HF mice treated with sh-HDAC3 + NC-antagomir. & *p* < 0.05 *vs.* HF mice treated with sh-HDAC3 + oe-NC. One-way ANOVA was utilized for comparisons among multiple groups, and Tukey’s post-hoc test was employed for intra-group pairwise comparison

Additionally, HE staining (Fig. 7b) and Masson staining (Fig. 7c) demonstrated that inhibition of HDAC3 brought about a reduction in cardiomyocyte size and CVF in HF mice, and additional treatment with miR-18a-antagomir or oe-ADRB3 counteracted this trend. TUNEL staining showed that the inhibition of HDAC3 also decreased the apoptosis in mouse heart tissue in response to sh-HDAC3, which could be reversed following treatment with miR-18a-antagomir or oe-ADRB3 (Fig. 7d). To conclude, these findings indicated that HDAC3 inhibition up-regulated miR-18a to reduce the ADRB3 expression, thereby alleviating HF in mice.

## Discussion

HF is a heterogeneous clinical characteristic of cardiac overload and injury accounting for high morbidity and mortality rates all over the globe [17]. Approximately half of the associated mortality with HF is exacerbated due fatal ventricular arrhythmias like ventricular tachycardia and ventricular fibrillation [18]. The primary cause of HF exacerbation is left ventricular dysfunction stems from myocardial interstitial fibrosis [19]. On the other hand, the hard-done work of our peers has revealed that miRs can function as viable biomarkers for HF due to their differential expression in HF [20]. In the current study, we set out investigate the underlying mechanism of miR-18a in the progression of HF, and the obtained findings elicited that HDAC3 radically-inhibited miR-18a to promote the ADRB3 expression, thereby, aggravating HF.

Initially, our findings revealed that miR-18a was poorly-expressed in HF mice and cardiomyocytes. Other researchers have uncovered close association between miR-18a and systemic ventricular contractility [21]. Moreover, down-regulated expression of miR-18a has also been previously documented in HF [8]. Furthermore, we discovered that over-expression of miR-18a brought about a suppressive effect on the fibrosis, hypertrophy and apoptosis of cardiomyocytes, as evidenced by reduced cMyb, MMP-9, Collagen 1, and TGF-β1 expressions. MMP-9 is known to be extensively-regulated in the process of fibrosis and immune dysregulation [22]. Also, TGF-β1 can fundamentally promote cardiac fibrosis, thereby significantly precipitating the progression of HF [23]. Meanwhile, the conventional hallmark of fibrosis is characterized by the accumulation of various fibrillar collagens, which principally serve as a marker of fibrosis, especially of Collagen 1 [24]. More notably, previous studies have demonstrated that miR-18a can diminish the expression of Collagen 1 via interleukin-23 [25]. Further in line with our findings, Yang et al. [26] demonstrated that the involvement of miR-18a in the signaling pathway of TGF-β1 by mediation of CCN2. Principally, miR-18a-5p impedes cardiac fibrosis by inhibiting the Notch2 pathway [27]. miR-18a-5p can radically constrain sub-pleural pulmonary fibrosis through targeting TGF-β receptor II [28]. Overall, our findings in conjunction with existing evidence indicate that miR-18a was poorly-expressed in HF, whereas over-expression of miR-18a reduced the fibrosis, hypertrophy, and apoptosis of cardiomyocytes in HF.

Furthermore, to investigate the underlying downstream mechanisms regulated by miR-18a, we performed a series of experiments to predict and verify the downstream target genes of miR-18a. Interestingly, our findings provided substantial evidence supporting ADRB3 as a direct target gene of miR-18a, wherein miR-18a inhibited the expression of ADRB3 in mouse cardiomyocytes. As a series of small, endogenous, non-coding RNAs, miRs possess the ability to negatively-regulate gene expressions by suppressing translation or facilitating the degradation of target mRNAs [29]. For instance, the regulation of miRs is known to be essential in the hypertension-induced arterial remodeling process via regulation of ADRB (1, 3) pathways [30]. In addition, miR-18a can explicitly target the IGFI factor to subsequently induce myotubes atrophy [31]. Additionally, our findings demonstrated that ADRB3 was highly-expressed in HF mice and cardiomyocytes. On the contrary, the receptor antagonist of ADRB3 has been previously shown to significantly improve pacing-induced HF, which is in accordance with our findings [16]. Besides, ADRB3 can also potentiate cardiomyocyte fibrosis, hypertrophy and apoptosis to further aggravate HF. Moreover, existing evidence suggests that ADRB3 immensely supports the metabolic processes of other cardiovascular and metabolic dysfunctions [32]. An existing study reported an overexpression of ADRB3 in canine models of atrial fibrillation, and that ADRB3 up-regulation elevated atrial myocyte apoptosis, fibrosis and atrial dilatation [33]. Meanwhile, a forced expression of ADRB3 exacerbated the progression of myocardial infarction in diabetic rats [34].

Studies have further shown that HDAC3 can induce the down-regulation of miR-18a and promote HF development [11, 13]. Consequently, we explored whether HDAC3 mediated HF via miR-18a by altering HDAC3, miR-18a and ADRB3 levels in HF cardiomyocytes, and found that HDAC3 was highly-expressed in HF mice and cardiomyocytes. What’s more, another study uncovered higher levels of HDAC (1, 2, 3, 4, 6) in the left ventricles of HF rats compared to healthy controls [35]. Similarly, markedly-elevated HDAC3 activity was previously noted in the hearts of diabetic mice [36]. The cardiomyocyte dysfunction of atrial fibrillation is further attributed to over-expression of Class I HDAC3 [37]. Besides, HDAC3 is known to promote cardiomyocyte fibrosis, hypertrophy and apoptosis by inhibiting miR-18a. In accordance with our findings, HDAC3 inhibition can augment the miR-130a expression in patients with spinal cord injuries [38]. A significant loss-of function of HDAC3 can also promote the miR-200a expression [39]. Further in line with our findings, up-regulation of HDAC3 was strongly associated with the down-regulation of miR-195 in hepatocellular carcinoma cells [40]. These findings implicitly highlight the ability of HDAC3 to down-regulate miR-18a. Also, the inhibition of HDAC3 has been previously shown to suppresses myocyte hypertrophy and alleviate LVHF in animals [13]. After instilling melatonin treatment, the cardio fibrosis along with the gene expressions of HDAC1 (1, 2, 3, 4, 6) are both alleviated [41]. Furthermore, inhibition of HDAC3 can improve cell viability and alleviate apoptosis in cerebral ischemia reperfusion injury mice in diabetic state [42]. Thus, with the supporting findings, we speculate that HDAC3 regulates the expression of miR-18a by binding to the promoter region of miR-18a to radically regulate the expression of its down-stream target gene ADRB3, thus coherently affecting the progression of HF.

## Conclusions

Altogether, the findings obtained in the current study highlight that HDAC3 inhibits miR-18a to increase the ADRB3 expression, thus aggravating HF by inducing fibrosis, hypertrophy and apoptosis of cardiomyocytes. Our discoveries might provide a viable target for the treatment of HF. However, additional studies are needed for further exploration in the clinical setting.

## Materials and methods

### Ethics statement

All study protocols were approved by the Medical and Clinical Research Ethics Committee of The Third Affiliated Hospital of Qiqihar Medical University, and were in compliance with *the Laboratory Animal Care and Use Guidelines* (NIH Publication No. 85-23, revised in 1996). Extensive efforts were made to minimize the suffering of the included animals.

### Animal model

A total of 150 healthy male C57Bl/6J mice (aged 10–12 weeks) were procured from the animal experiment center of The Third Affiliated Hospital of Qiqihar Medical University. The obtained mice were housed in a specific-pathogen free (SPF)-level purification space under a photoperiod of 12/12 h light/dark at 22 ± 3 °C, with relative humidity ranging from 40 to 70%. All mice had ad libitum access to food and water. Ten mice were randomly selected for sham surgery. The remaining 140 mice were reserved for establishing HF models. HF mice models after myocardial infarction were established by ligation of the left coronary artery at the lower left atrial appendage between the left atrial appendage and the pulmonary artery cone of the mice [43]. Meanwhile, only the thread was used without ligation in the sham-operated mice. Ultrasound electrocardiography was subsequently performed 4 weeks after surgery using a cardiac ultrasound system (SonoAce X8, Shanghai Poly Medical Instruments Co., Ltd., Shanghai, China). The success rate of modeling was calculated to be 89.29% (125/140). Animals that failed in model establishment were replaced with desirable ones.

### Mouse grouping

After successful model establishment, the oligonucleotides, plasmids, and transfected plasmid complexes (miR-18a-agomir, short hairpin [sh]-ADRB3, over-expression [oe]-ADRB3, sh-HDAC3, miR-18a-antagomir, negative control [NC]-agomir, sh-NC, oe-NC, and NC-antagomir) were diluted according to the provided protocols of En-transter™-in vivo and injected into the mice via the tail vein [44]. Subsequently, miR-18a-antagomir and NC-antagomir were injected into the mice at a dosage of 80 mg/kg, while miR-18a-agomir and NC-agomir were injected into the mice at a dosage of 100 nmol/kg. Meanwhile, interference or over-expression lentivirus was injected at a dose of 100–300 uL per mouse (virus titer was about 108–109 PFU/TU). Control mice were instilled an equivalent amount of solvent for 3 days [45]. All cohesive procedures including vector construction, sequencing and identification, virus packaging and titer detection were commissioned to the Shanghai Genechem Co., LTD. (Shanghai, China). miR-18a-agomir (UAAGGUGCAUCUAGUGCAGAUAG), miR-18a-antagomir (AUUCCACGUAGAUCACGUCUAUG), NC-agomir (micrON agomir NC#22), and NC-antagomir (micrOFF antagomir NC#24).

### Mouse cardiomyocyte model

The ventricles of C57Bl/6J suckling mice (aged 2–3 days) were harvested under aseptic conditions, and rinsed 3 times with Hank's solution (pH = 7.2–7.4). The ventricles were then dissected and trypsinized for 10 min at 37 °C and for 30 min at 37 °C. Next, the tissues were rinsed 3 times with Hank's solution and then pipetted until complete cells dispersion. The cell concentration was subsequently adjusted to 2 × 10^6^ cells/mL using Dulbecco's modified Eagles Medium (DMEM) containing 20% fetal bovine serum (FBS), and transferred to a cell culture flask. The cell culture flask was incubated for 4 h at 37 °C with 5% CO_2_. The adherent cells were then discarded and the survival rate of the cells was detected using trypan blue staining. When the cell survival rate was over 95%, the cell suspension was transferred to a 96-well plate (100 μL per well) and placed in a 5% CO_2_ incubator at 37 °C for 7 days. HF cell models were induced with the addition of H_2_O_2_. Briefly, H_2_O_2_ was added to 10% serum culture medium to attain a final concentration of 100 m/L, and cultured for 30 min to facilitate cell model construction. Afterwards, the cells were seeded in a 6-well plate 24 h prior to transfection to attain 70% cell confluence. Cells were transfected with the respective carriers, which were as follows: NC-mimic, miR-18a-mimic, sh-NC, sh-ADRB3, oe-NC, oe-ADRB3, sh-HDAC3, miR-18a-inhibitor and NC-inhibitor, in strict accordance with the provided manuals of the Lipofectamine 2000 kit (11668019, Thermo Multiskan MK3, Thermo Fisher Scientific, Waltham, MA, USA). After 6 h of transfection, the culture medium was replaced, and the cells were collected for subsequent experimentation after another 48 h of culture. The aforementioned carriers were sourced from Shanghai Genechem Co., LTD.

### Mouse echocardiography

All mice were intraperitoneally anesthetized with 3% sodium pentobarbital (P3761, Sigma-Aldrich, St. Louis, MO, USA). The chests of the mice were shaved and the mice were fixed onto wooden boards in supine position. Subsequently, a color ultrasound diagnostic apparatus (SSI-5000, Shandong Shukang Hengtong Science & Trade Co., Ltd., Shandong, China) was adopted to measure the left ventricular posterior wall dimension (LVPWD), interventricular septal dimension (IVSD), left ventricular end diastolic dimension (LVEDD), left ventricular end systolic diameter (LVESD), left ventricular ejection fraction (LVEF) and left ventricular fractional shortening (LVFS), respectively.

### Detection of hemodynamic parameters in mice

The mice were intraperitoneally anesthetized and fixed in supine position on the operating table. Subsequently, the left ventricular systolic pressure (LVSP), left ventricular end diastolic pressure (LEVDP), heart rate (HR), left chamber pressure rise rate (+ dp/dt), left chamber pressure drop rate (−dp/dt) were simultaneously recorded using a multi-channel physiological recorder (p3 plus, Beijing B&E TEKSYSTEMS Co., Ltd., Beijing, China).

### Hematoxylin and eosin (HE) staining

Myocardial tissue samples of the mice were rinsed with physiological saline, and then fixed with 4% paraformaldehyde for 30–50 min. Next, the samples were dehydrated, cleared, paraffin-embedded, and then sliced periodically. The sections were subsequently flattened, placed on a glass slide, and dried at 45 °C, followed by dewaxing and hydration. Afterwards, the sections were stained with hematoxylin for 5 min, differentiated with 1% hydrochloric acid ethanol for 3 s, and finally stained with 5% eosin for about 3 min. After dehydration, clearance and mounting, the sections were finally observed under a microscope.

### Masson staining

The paraffin sections of the mouse myocardial tissues were oven baked at 65 °C for 3 h, followed by dewaxing and dehydration. Next, the sections were individually immersed in 10% trichloroacetic acid and 10% potassium dichromate solutions for 40 min each. After a regimen of 8-min staining with hematoxylin (PT001, Shanghai Bogoo Biotechnology. Co., Ltd., Shanghai, China), the sections were immersed in 1% ponceau S (HL12202, Shanghai Haling Biotechnology Co., Ltd, Shanghai, China) and 1% magenta mixed solution (HPBIO-SJ820, Shanghai HePeng Biotech., Co., Ltd., Shanghai, China) for 40 min. The reaction was terminated with the addition of 1% glacial acetic acid (first) and 1% molybdic acid solution (post). Later, the sections were routinely dehydrated, blocked using a transparent neutral resin and then observed. Positive results were estimated as follows: basement membrane and collagen fibers stained in blue or green, immune complexes stained in red, and the nuclei stained in blue-brown. The sections were subsequently photographed under a polarizing microscope, with 5 randomly chosen fields for each section. The Image-Pro plus 5.1 image analysis software (Media Cybernetics, Silver Springs, MD, USA) was then adopted for image analysis and to subsequently calculate the collagen volume fraction (CVF), which was as follows: CVF (%) = collagen area/full field area × 100%.

### Terminal deoxynucleotidyl transferase (TdT)-mediated dUTP nick-end labeling (TUNEL) staining

The aforementioned paraffin sections were routinely dewaxed and dehydrated. Next, the sections were immersed in 3% H_2_O_2_ methanol solution for 30 min at room temperature to terminate the peroxidase activity. Subsequently, the proteinase K working solution was supplemented and the sections were incubated at 37 °C for 30 min. The sections were then immersed in 0.1% TrionX-100 and 0.1% sodium citrate solution and subjected to recovery at room temperature for 5 min. The freshly prepared TUNEL reaction mixture (prepared according to the provided TUNEL kit instructions) was then supplemented, followed by 90-min culture and the sections until room temperature was restored. Afterwards, 5% bovine serum albumin (BSA) blocking solution was supplemented to incubate the sections for 20 min at room temperature in a wet box. Drop-transformed Plant peroxidase (POD) solution was then added to the culture sections for 30 min at 37 °C. The sections were colored at room temperature using diaminobenzidine. Observation was conducted from five randomly selected fields from each section, and the number of TUNEL-positive cells (with brown-yellow particles in the nucleus) in each field of view was measured under a 400- or 200-fold microscope with the help of the BI-2000 image analysis system.

### Immunohistochemical staining

The aforementioned paraffin sections were treated by boiling twice with a 5-min interval and then restored to room temperature. Next, the sections were rinsed with PBS and sealed with normal goat serum sealing solution. In the experimental process, the known positive sections of the HCC sections were adopted as positive control, while the immunoglobulin G (IgG) replaced the primary antibody as the NC, and the Histostain™ SP-9000 immunohistochemical staining kit (Zymed lab. Inc., San Francisco, California, USA) was employed for staining. Primary antibody ADRB3 (ab94506, Abcam, Cambridge, UK)/HDAC3 (ab32369, Abcam) was added and incubated at 4 °C overnight. After rewarming and rinsing with PBS, the goat anti rabbit secondary antibody (ab6721, Abcam) was added and incubated at 37 °C for 30 min. After repeated PBS rinses, the horseradish labeled working solution was added. DAB was then added to facilitate the color development for 5 to 10 min. The staining time was adjusted under the microscope. After counterstaining with hematoxylin for 1 min, the sections were sealed, documented and stored [46].

### In situ hybridization

The miR-18a expression in the myocardial tissues was detected with the FISH technique using an in-situ hybridization kit (MK3257, Wuhan Boster Biological Technology Co., Ltd., Hubei, China). The paraffin sections were routinely dewaxed with water and rinsed twice with PBS. Next, the sections were treated with the proteinase K diluted solution for 20 min and pre-denaturation solution for 8 min. After undergoing sealing with the pre-hybridizing solution at 37 °C, the sections were hybridized overnight with the digoxin labeled miR-18a probes (Wuhan Boster Biological Technology Co., Ltd.) 37 °C. Subsequently, the sections were rinsed with hybridization solution in conditions devoid of light at 42 °C, and the hybridization region was stained with 4,6-diaminol-2-phenylindole (DAPI) (Sigma-Aldrich). Finally, the sections were fixed on slides under light-proof conditions and observed under a laser confocal microscope [47].

### Reverse transcription-quantitative polymerase chain reaction (RT-qPCR)

A total of 100 μL of the tissue homogenate or cells from each group were placed in a reaction tube to isolate the total RNA content with 1 mL of the TRIzol reagent (15596-018, Beijing solarbio science & technology co. ltd., Beijing, China). Subsequently, 2 μg RNA was obtained for cDNA synthesis using the TaqMan reverse transcription reagent (Roche, Basel, Switzerland) at 42 °C for 50 min, and then PCR (50 μL reaction system) was conducted to amplify the target gene fragment. The primers used in the PCR were synthesized by Sigma-Aldrich (Table 7). The amplification conditions were as follows: pre-denaturation at 94 °C for 5 min, 30 cycles of denaturation at 94 °C for 45 s, annealing at 55 °C for 45 s and extension at 72 °C for 45 s, and extension at 72 °C for another 10 min. The relative expression levels of ADRB3 and HDAC3 or miR-18a were normalized to those of glyceraldehyde-3-phosphate dehydrogenase (GAPDH) or U6 and calculated on the basis of the 2^−ΔΔCt^ method for CT value estimation [48].

**Table 7:** Primer sequences for RT-qPCR

Gene	Forward primer (5′-3′)	Reverse primer (5′-3′)
miR-18a	TAAGGTGCATCTAGTGCAGATAG	GCGAGCACAGAATTAATACGAC
ADRB3	CGCCTTCAACCCGGTCATCTACTG	GGTGGACTCTGCCTGGCTTCAAC
HDAC3	GACATGTGCCGCTTCCATTC	CTGGCTGGAAAAGGTGCTTG
U6	GCATGACGTCTGCTTTGGA	CCACAATCATTCTGCCATCA
GAPDH	AGGTCGGTGTGAACGGATTTG	TGTAGACCATGTAGTTGAGGTCA

### Western blot assay

A total of 1 mL of cell lysate was supplemented with 100 μL of the heart tissue homogenate or cardiomyocytes for a regimen of 30-min lysing at 4 °C. The supernatant was then harvested as a protein extract with 20 min-centrifugation at 12,000 r/min and 4 °C. Sodium dodecyl sulfate–polyacrylamide gel electrophoresis separation gel (10%) and concentrated gel (5%) were prepared for electrophoretic separation of proteins. The obtained protein on the gel was electroblotted onto a nitrocellulose membrane, which underwent an overnight blockade at 4 °C with 5% skim milk powder. Then, the primary anti-rabbit antibodies to HDAC3 (ab32369, dilution ratio of 1: 5000, Abcam), ADRB3 (SAB4500584, dilution ratio of 1: 800, Sigma-Aldrich), cMyb (AV38611, dilution ratio of 1: 1000, Sigma-Aldrich), matrix metalloproteinase 9 (MMP-9) (ab38898, dilution ratio of 1: 1000, Abcam), Collagen 1 (ab21286, dilution ratio of 1: 1000, Abcam), and TGF-β1 (ab92486, dilution ratio of 1: 1000, Abcam) were added to probe the membrane overnight at 4 °C. Subsequently, the goat anti-rabbit IgG (ab6721, dilution ratio of 1: 5000, Abcam) complexed to horseradish peroxidase was supplemented, followed by a 1-h regimen of re-probing the membrane at room temperature. Next, developing solution was added to facilitate the development of the membrane. The quantitative gray value of each band was analyzed using the Quantity One software, and the relative quantitative analysis of the target protein was estimated with anti-rabbit GAPDH (ab8245, dilution ratio of 1:2000, Abcam) as the loading control.

### Dual luciferase reporter assay

The target site sequence [wild type (WT)] of the 3′-untranslated regions (3′-UTR) region of ADRB3 mRNA and the sequence [mutant (MUT)] after site-directed mutagenesis of the WT target site were artificially synthesized. Subsequently, the pmiR-RB-REPORTTM plasmid was digested using restriction enzymes. The artificially synthesized target gene fragments WT and MUT were then implanted in the pmiR-RB-REPORTTM vector (RiboBio Co., Ltd., Guangzhou, China), respectively, and the empty plasmid was transfected as control. The correct luciferase reporter plasmids WT and MUT were sequenced for subsequent transfection. Next, the vectors containing MUT and WT were co-transfected into the HEK293T cells with the NC mimic or miR-18a mimic, respectively. After 48 h of transfection, the cells were lysed and centrifuged for 3 to 5 min, after which the supernatant was collected. Relative luciferase units (RLU) were assayed using Renilla Luciferase Assay kits (YDJ2714, Shanghai Yuduo Biotech Co., Ltd., Shanghai, China). A dual luciferase reporter assay system (Promega Co, Madison, WI, USA) was adopted for analysis with the firefly luciferase as the loading control.

### Flow cytometry assay

After 48 h of cell transfection, the medium was rinsed with phosphate buffer saline (PBS). The cells were then detached with 0.25% trypsin (without ethylene diamine tetra acetic acid) and centrifuged, followed by discarding of supernatant. According to the protocol of the Annexin-V-FITC Apoptosis Detection kit (556547, Shanghai Shuojia Biotechnology Co., Ltd., Shanghai, China), the Annexin-V/PI dye solution was formulated with a combination of Annexin-V-fluorescein isothiocyanate (Annexin-V-FITC), propidium iodide (PI), 4-(2-hydroxyethyl)-1-piperazineëthanesulfonic acid (HEPES) buffer solution in the ratio of 1: 2: 50. The cells were resuspended using the Annexin-V/PI dye solution (1 × 10 ^6^ cells/100 μL), shaken, and incubated for 15 min at room temperature. Next, 1 mL of HEPES buffer was supplemented and mixed completely. Lastly, apoptosis was measured using a flow cytometer (Bio-Rad ZE5, Bio-Rad, Richmond, Cal, USA).

### Cardiomyocyte imaging

Transfected cells were seeded on 35 mm glass dishes (MatTek Corporation, Ashland, MA, USA) and incubated with calcium indicator fluo-4AM (5 μmol/L; Invitrogen, Eugene, OR, USA) for 20 min at 37 °C. Next, the cells were rinsed with Hanks' balanced salt solution, and the medium and petri dish were replaced. A closed and thermally controlled (37 °C) stage-top incubator (Tokai Hit Co., Shizuoka-ken, Japan) was inserted over an inverted Nikon TiE fluorescence microscope (Nikon Instruments, Tokyo, Japan) electric bench equipped with a × 60 oil-immersed optical lens (CFI PlanFluor, NA 1.43; Nikon Instruments) and NIS Elements software. A Lumencor diode-pumped light engine (SpectraX; Lumencor Inc., Beaverton, OR, USA), ET-GFP filters (Chroma Technology Corporation, Bellows Falls, VT, USA) and ORCA-Flash 4.0 sCMOS cameras (Hamamatsu Corporation, Bridgewater, NJ, USA) were used for comprehensive testing. Data was collected every 3 min within 30 min.

### Statistical analysis

Statistical analyses were performed using the SPSS 21.0 software (IBM Corp. Armonk, NY, USA), and the measurement data was depicted as mean ± standard deviation. Independent sample *t*-test was adopted to compare the data between two groups. One-way analysis of variance (ANOVA) was utilized for comparisons among multiple groups, and Tukey’s post-hoc test was employed for intra-group pairwise comparison. All tests were bilateral, and a value of *p* < 0.05 was indicative of statistical significance.

## Supplementary Information

**Additional file 1: Fig. S1. **Cardiomyocyte imaging. **A**, Cardiomyocyte size observed by cardiomyocyte imaging (40 ×). **B**, Cardiomyocyte imaging of cardiomyocyte size (40 ×).** C**, Cardiomyocyte imaging of cardiomyocyte size (40 ×).

**Additional file 2:** **Fig. S2. **HDAC3 downregulation attenuates HF by suppressing miR-18a-targeted ADRB3 in mice. **A**, RT-qPCR detection of the expression pattern of HDAC3 in myocardial tissues of HF mice normalized to GAPDH. **B**, Western blot analysis of the expression pattern of HDAC3 in myocardial tissues normalized to GAPDH. **C**, Immunohistochemistry and In situ hybridization of the expression patterns of HDAC3, ADRB3 and miR-18a in the mouse cardiomyocytes. * *p *< 0.05 *vs.* HF mice treated with sh-NC. #* p *< 0.05 *vs.* HF mice treated with sh-HDAC3 + NC-antagomir. &* p *< 0.05 *vs.* HF mice treated with sh-HDAC3 + oe-NC. The data results were measurement data and expressed as mean ± standard deviation. n = 10. One-way ANOVA was utilized for comparisons among multiple groups, and Tukey’s post-hoc test was employed for intra-group pairwise comparison.

## Data Availability

The datasets generated and/or analyzed during the current study are available from the corresponding author on reasonable request.

## Abbreviations

BSA: Bovine serum albumin
CVF: Collagen volume fraction
HF: Heart failure
HR: Heart rate
IVSD: Interventricular septal dimension
LEVDP: Left ventricular end diastolic pressure
LVEDD: Left ventricular end diastolic diameter
LVEF: Left ventricular ejection fraction
LVESD: Left ventricular end systolic diameter
LVFS: Left ventricular fractional shortening
LVPWD: Left ventricular posterior wall dimension
LVSP: Left ventricular systolic pressure
SPF: Specific-pathogen free
